# Vitamin D status in patients with arthritis and osteoporosis: a comparative analysis of inpatient and outpatient cohorts

**DOI:** 10.3389/fendo.2026.1803333

**Published:** 2026-09-15

**Authors:** Ruoxuan Wang, Wen Zheng, Yali Wen, Yishan Li, Shoujun Cheng, Weihong Zhou, Yang Qin, Haodang Chang, Weihao Meng, Yinhe Wang

**Affiliations:** 1 Department of Clinical Chinese Medicine, Nanjing University of Chinese Medicine, Nanjing, Jiangsu, China; 2 Department of Geriatrics, Nanjing Drum Tower Hospital, The Affiliated Hospital of Nanjing University Medical School, Nanjing, Jiangsu, China; 3 Translational Medicine Research Center, Children’s Hospital of Nanjing Medical University, Nanjing, China; 4 Department of Translation Studies, Army Engineering University of PLA, Nanjing, China; 5 Department of Clinical Laboratory, Nanjing Drum Tower Hospital, The Affiliated Hospital of Nanjing University Medical School, Nanjing, Jiangsu, China; 6 Department of Health Management Centre, Drum Tower Hospital Affiliated to Nanjing University Medical School, Nanjing, Jiangsu, China; 7 Department of Surgery, Nanjing University of Chinese Medicine, Nanjing, Jiangsu, China; 8 Department of Orthopaedics, Wuxi 9th People’s Hospital Affiliated to Soochow University, Wuxi, China; 9 Department of Orthopedics, Clinical College, Nanjing Drum Tower Hospital, Nanjing, Jiangsu, China

**Keywords:** 25-hydroxyvitamin D, arthritis, bone health, inpatients, osteoporosis, outpatients, risk factors, vitamin D deficiency

## Abstract

**Background:**

Vitamin D deficiency is a global health issue linked to musculoskeletal disorders, including osteoporosis and arthritis. Despite its critical role in bone and immune health, large-scale comparisons of vitamin D status between outpatient and inpatient populations remain limited.

**Objective:**

This study aimed to compare vitamin D levels in patients with and without arthritis or osteoporosis across outpatient and inpatient settings and evaluate their associations with clinical outcomes.

**Methods:**

We performed a retrospective cross-sectional analysis of 37800 adults treated at the Department of Orthopedics in Nanjing Drum Tower Hospital from 2015 to 2020. Serum 25(OH)D concentrations were categorized as normal, low, or deficient. Patients were stratified by diagnosis (arthritis and osteoporosis) and care setting (outpatient vs. inpatient). Statistical comparisons were made using t-tests, Chi-square tests, and log-binomial regression adjusted for age, sex, the alternate musculoskeletal diagnosis, and osteocalcin. Diabetes mellitus was only consistently available for inpatients and was therefore handled as a descriptive inpatient characteristic rather than in the outpatient characteristic and regression models.

Adjusted relative risks (RRs) for arthritis and osteoporosis were estimated according to vitamin D status, with normal vitamin D as the reference category, using log-binomial regression adjusted for age, sex, osteocalcin, and the alternate musculoskeletal diagnosis where applicable.

**Results:**

Suboptimal vitamin D status (low or deficient levels) was widespread across both settings, affecting 52% of outpatients and 41% of inpatients. The overall mean 25(OH)D was 26.25 ± 7.14 ng/mL in outpatients and 27.38 ± 6.58 ng/mL in inpatients. Among outpatients, those with arthritis exhibited higher deficiency rates (16.2% vs. 13.6% in non-arthritis) and significantly elevated arthritis risks (RR ranging from1.24 to 1.32). Among inpatients, however, the arthritis analysis was limited by the small number of cases (n=176) and wide confidence intervals (RR for deficiency=1.08, 95% CI 0.65-1.70), so the finding is inconclusive and does not support the association. Deficiencies were particularly pronounced in osteoporosis cases: 26.3% of osteoporotic outpatients were deficient (vs.12.8% controls; RR = 2.79), while inpatients with osteoporosis also showed higher deficiency (13.7% vs. 10.2%; RR = 1.50), with both differences statistically significant (p<0.001).

**Conclusion:**

Vitamin D deficiency was common, particularly among osteoporosis patients in outpatient settings. Associations were strongest for outpatient osteoporosis, while the inpatient arthritis estimates were limited by the small number of cases. These findings suggest that targeted assessment of vitamin D status in patients with osteoporosis or arthritis, rather than universal screening, may help identify individuals at higher risk of deficiency and related musculoskeletal complications.

## Introduction

Vitamin D is a critical nutrient for musculoskeletal and immune health. Widespread vitamin D deficiency has been recognized as a global public health issue (1). A recent pooled analysis of 7.9 million people from 81 countries found that approximately 15.7% of individuals have severe vitamin D deficiency (25-hydroxyvitamin D [25(OH)D] <30 nmol/L) and nearly 47.9% have insufficient levels (<50 nmol/L) (2). Vitamin D deficiency in adults can lead to osteomalacia and contributes to osteoporosis, thereby increasing the risk of fragility fractures (3, 4). Given its role in calcium and phosphate metabolism, low vitamin D impairs bone mineralization (5, 6) and is associated with a higher prevalence of osteoporosis (7). For these reasons, maintaining adequate vitamin D is a cornerstone of bone health, especially in older adults.

Patients with chronic musculoskeletal disorders, such as arthritis and osteoporosis, are often at high risk for vitamin D insufficiency (8). Arthritis comprises a heterogeneous group of disorders, including inflammatory conditions such as rheumatoid arthritis (RA) (9) and psoriatic arthritis (10), as well as degenerative conditions such as osteoarthritis (11). Vitamin D deficiency has been reported in both inflammatory and non-inflammatory forms of arthritis, although the underlying mechanisms and strength of association may differ. Among inflammatory arthritides, RA is one of the most extensively studied diseases, with reports indicating that up to 84% of patients have suboptimal vitamin D levels, and lower vitamin D concentrations are associated with increased disease activity and pain sensitivity (12, 13).

Osteoporosis is a systemic metabolic bone disease characterized by reduced bone mineral density (BMD), deterioration of bone microarchitecture, and an increased risk of fragility fractures (14). Its prevalence rises markedly with advancing age, particularly among postmenopausal women, in whom estrogen deficiency accelerates bone loss and compromises skeletal integrity. Vitamin D plays an essential role in maintaining skeletal health by regulating calcium and phosphate homeostasis and promoting normal bone remodeling. Similarly, in osteoporosis, vitamin D insufficiency is widely prevalent and exacerbates bone loss (15). Postmenopausal women with osteoporosis frequently have suboptimal 25(OH)D levels; for example, a large multinational study found that less than one-quarter of postmenopausal women with osteoporosis had vitamin D sufficiency (>30 ng/mL) (16). Ensuring sufficiency is known to improve calcium absorption and the efficacy of osteoporosis treatments (17, 18). Indeed, vitamin D and calcium supplementation is recommended as standard of care in osteoporosis management to support bone density and reduce fracture risk (19, 20).

Despite the known importance of vitamin D, there is a need to better characterize how vitamin D status varies among different patient subgroups and clinical settings. In particular, comparing ambulatory (outpatient) individuals to hospitalized (inpatient) patients can illuminate potential gaps in care or differences in risk profiles. This study aimed to assess and compare vitamin D levels in patients with and without arthritis and osteoporosis in both outpatient and inpatient settings. By analyzing a large hospital-based cohort, we sought to determine the significance of vitamin D deficiency among these clinical subgroups and to discuss the public health implications of our findings for improving patient outcomes.

## Methods

### Study design and population

We conducted a retrospective cross-sectional study using de-identified health records of patients treated between 2015 and 2020 in the orthopedics department of Nanjing Drum Tower Hospital, a large tertiary care center.

The inclusion criteria were as follows: Adult patients aged 18 years or older with at least one recorded measurement of serum 25-hydroxyvitamin D [25(OH)D] during the study period were eligible. Patients were included if they were either outpatients attending hospital clinics or inpatients admitted to the tertiary care center. Within these groups, individuals were classified according to the presence or absence of a documented diagnosis of arthritis (including rheumatoid arthritis (21), osteoarthritis (22), psoriatic arthritis (23), or other inflammatory/degenerative joint disease (24–26)) based on established clinical diagnostic criteria and corresponding ICD-10 codes: RA (M05, M06), OA (M15–M19), PsA (L40.5, M07)in the medical record. Osteoporosis was identified from dual-energy X-ray absorptiometry measurements of the femur, hip, or lumbar spine (L1–L4) and from documented fragility fractures. Participants were categorized as having normal bone mass (T-score ≥−1), osteopenia (T-score >−2.5 and <−1), or osteoporosis (T-score ≤−2.5 or a qualifying fragility fracture). For the binary analyses, participants with normal bone mass or osteopenia were classified as not having osteoporosis (27, 28). For women who have not yet reached menopause and men younger than 50, bone density levels were evaluated utilizing Z-scores that correspond with the same ethnic background. Furthermore, bone fractures that were linked to abnormal bone metabolism were also taken into account. Demographic information (age, sex) and key clinical data, including comorbidities such as diabetes mellitus in inpatients were also obtained. Osteocalcin was included as a covariate to account for differences in bone turnover, which may influence both bone and joint outcomes.

The exclusion criteria were: Patients with conditions likely to confound vitamin D metabolism or bone health interpretation, including metabolic bone diseases other than osteoporosis (such as osteomalacia not due to vitamin D deficiency, Paget’s disease of bone, or hyperparathyroidism). We also excluded individuals with severe renal impairment (chronic kidney disease stage 4–5 or end-stage renal disease requiring dialysis), advanced liver disease, or malabsorptive disorders (celiac disease, inflammatory bowel disease with malabsorption, or gastric bypass surgery). Patients with active malignancy affecting bone metabolism (multiple myeloma or bone metastases), those on long-term medications with profound effects on vitamin D metabolism (such as certain anticonvulsants), and those with incomplete laboratory or demographic data were also excluded.

The final dataset comprised two cohorts of adult patients: outpatients (n = 20342) who visited hospital clinics and inpatients (n = 17458) who were admitted to the hospital. Within each cohort, patients were classified by arthritis and osteoporosis status defined above (29, 30). For patients with both outpatient and inpatient records, cohort assignment was based on the setting of the included 25(OH)D measurement. To avoid duplicate inclusion, only the first available 25(OH)D measurement and corresponding clinical information were analyzed.

The diagnostic data structure differed between the outpatient and inpatient cohorts. In the outpatient records, only one diagnosis was available for each patient. Arthritis and osteoporosis were therefore mutually exclusive in the outpatient dataset because of the recording structure, although the two conditions may have coexisted clinically. In contrast, inpatient records could contain up to four diagnoses, allowing some patients with both arthritis and osteoporosis to be identified.

### Measurements

Blood samples were analyzed for 25-hydroxyvitamin D (25[OH]D) levels as part of routine care. Serum 25(OH)D is reported in ng/mL and was used as the indicator of vitamin D status. In addition, serum osteocalcin (a marker of bone formation) was measured in patients as part of bone health evaluation. In our study, both vitamin D and osteocalcin measurements were performed as part of routine clinical testing rather than research-specific laboratory assays. For analysis, vitamin D status was categorized into three groups according to established clinical thresholds for serum 25(OH)D concentrations: normal (sufficient, ≥30 ng/mL), low (insufficient, ≥20 and <30 ng/mL), and deficit (deficient, <20 ng/mL) (31–33). We note that different guidelines define sufficiency variably; in this study’s context “Normal” vitamin D refers to levels considered adequate in standard clinical practice, while “Deficit” indicates a marked deficiency likely to require intervention. Patient demographic data (age, sex) were recorded for both cohorts; diabetes mellitus was recorded for inpatients only, as described above.

### Statistical analysis

We stratified the analysis by care setting (outpatient vs. inpatient). Within each setting, we compared patients with vs. without arthritis, and separately compared those with vs. without osteoporosis. Continuous variables (such as 25(OH)D concentration and age) were summarized as mean and standard deviation (SD) if approximately normally distributed, or median and interquartile range (IQR) if skewed (osteocalcin levels were skewed and thus summarized as medians). Categorical variables (such as vitamin D status categories and sex) were summarized as counts and percentages. Group comparisons were performed using Student’s *t*-test for mean differences (or Wilcoxon rank-sum test for non-normal data) and Chi-square test for proportions. A two-tailed *p*-value < 0.05 was considered statistically significant. Less than 5% of patients had missing data in the study variables; therefore, these cases were excluded from the analysis, and no imputation methods were applied.

Additionally, we estimated adjusted relative risks (RRs) for arthritis and osteoporosis comparing low and deficient vitamin D status with normal vitamin D status, using log-binomial regression models adjusted for potential confounders (age, sex, osteocalcin, and the alternate diagnosis where appropriate). Diabetes mellitus was not included in the primary regression models because it was not consistently available for outpatients and the primary models were specified to be comparable across outpatient and inpatient settings. Inpatient diabetes mellitus is therefore only presented descriptively.

To address potential clinical overlap and incomplete capture of individual diagnoses, we conducted an additional analysis using a composite musculoskeletal outcome, defined as any recorded diagnosis of arthritis or osteoporosis. For outpatients, the composite represented the union of the two recorded diagnoses. For inpatients, the composite was defined using any arthritis or osteoporosis diagnosis recorded among the available diagnosis fields. Adjusted RRs for the composite outcome across vitamin D categories were estimated using log-binomial regression adjusted for age, sex, and osteocalcin. The alternate diagnosis was not included as a covariate because it formed part of the composite outcome.

Results are presented with *p*-values indicating the significance of differences between groups, and 95% confidence intervals (CI) for relative risks where applicable. Because the study is mainly exploratory and descriptive, p-values were not adjusted for multiple descriptive comparisons; instead, they should be interpreted alongside effect sizes, confidence intervals, and the consistency of findings.

Data handling and statistical analyses were conducted using R version 4.5.0 (R Foundation for Statistical Computing). The study was conducted in accordance with ethical standards and was approved by the Ethics Committee of Nanjing Drum Tower Hospital (Approval No. 2024-641).

## Results

### Cohort characteristics

A total of 20342 outpatients and 17458 inpatients were included in the analysis. The overall mean age was 42.11 ± 16.04 years for outpatients (**Table 1**) and 57.78 ± 16.66 years for inpatients (**Table 2**). Women represented 81.2% of the outpatient cohort and 43.1% of the inpatient cohort. In the outpatient group, 2421 patients (11.9%) had an arthritis diagnosis and 1717 (8.4%) had osteoporosis (**Table 1**). In the inpatient group, 176 patients (1.0%) had arthritis noted and 1427 (8.2%) had osteoporosis (**Table 2**). Diagnostic overlap could be evaluated only in the inpatient cohort because multiple diagnoses were available. Among the 176 inpatients with arthritis, 48 patients (27.3%) also had osteoporosis. Conversely, 48 of the 1,427 inpatients with osteoporosis (3.4%) had a recorded arthritis diagnosis (**Table 2**). Comparable overlap could not be determined in the outpatient cohort because only one diagnosis was recorded for each patient. The markedly lower proportion of arthritis among inpatients likely reflects the nature of hospital admissions, where chronic arthritis alone seldom necessitates admission (whereas osteoporosis-related fractures could). As expected, patients with osteoporosis were significantly older on average than those without, in both settings (*p* < 0.001). Likewise, the osteoporosis subgroup was predominantly female (about 84–88% female in those with osteoporosis vs. ~39–81% in those without, *p* < 0.001), consistent with the epidemiology of postmenopausal osteoporosis (34).

**Table 1:** Characteristics of outpatients with arthritis and osteoporosis.

Variable	All	Arthritis			Osteoporosis		
		No	Yes	p-value^*^	No	Yes	p-value^*^
Sample size, n (%)	20342 (100.0)	17921 (88.1)	2421 (11.9)		18625 (91.6)	1717 (8.4)	
Sex, n (%)				0.636			<0.001
Female	16527 (81.2)	14551 (81.2)	1976 (81.6)		15077 (81.0)	1450 (84.4)	
Male	3815 (18.8)	3370 (18.8)	445 (18.4)		3548 (19.0)	267 (15.6)	
Age, mean (SD)	42.11 (16.04)	41.15 (15.95)	49.20 (14.89)	<0.001	40.10 (14.71)	63.93 (13.52)	<0.001
Vitamin D, mean (SD)	26.25 (7.14)	26.34 (7.10)	25.60 (7.42)	<0.001	26.50 (6.97)	23.55 (8.34)	<0.001
Vitamin D level, n (%)				<0.001			<0.001
Normal	9760 (48.0)	8737 (48.8)	1023 (42.3)		9202 (49.4)	558 (32.5)	
Low	7738 (38.1)	6733 (37.6)	1005 (41.5)		7031 (37.8)	707 (41.2)	
Deficit	2838 (14.0)	2445 (13.6)	393 (16.2)		2386 (12.8)	452 (26.3)	
Osteocalcin, median [IQR]	15.10 [10.48, 21.95]	15.79 [11.45, 22.42]	14.96 [10.29, 21.88]	0.027	17.16 [11.66, 24.14]	13.75 [9.80, 19.99]	<0.001

* Student’s t-test or Wilcoxon rank-sum test were used for continuous variables, and Chi-squared test was used for categorical variables.

IQR: interquartile range.

**Table 2:** Characteristics of inpatients with arthritis and osteoporosis.

Variable	All	Arthritis			Osteoporosis		
		No	Yes	p-value^*^	No	Yes	p-value^*^
Sample size, n (%)	17458 (100.0)	17282 (99.0)	176 (1.0)		16031 (91.8)	1427 (8.2)	
Sex, n (%)				<0.001			<0.001
Female	7531 (43.1)	7396 (42.8)	135 (76.7)		6279 (39.2)	1252 (87.7)	
Male	9927 (56.9)	9886 (57.2)	41 (23.3)		9752 (60.8)	175 (12.3)	
Age, mean (SD)	57.78 (16.66)	57.70 (16.67)	65.32 (14.65)	<0.001	56.75 (16.55)	69.31 (13.20)	<0.001
Osteoporosis, n (%)				<0.001			
No	16031 (91.8)	15903 (92.0)	128 (72.7)				
Yes	1427 (8.2)	1379 (8.0)	48 (27.3)				
Arthritis, n (%)							<0.001
No	17282 (99.0)				15903 (99.2)	1379 (96.6)	
Yes	176 (1.0)				128 (0.8)	48 (3.4)	
Diabetes mellitus, n(%)				<0.001			<0.001
No	8777 (50.3)	8636 (50.0)	141 (80.1)		7662 (47.8)	1115 (78.1)	
Yes	8681 (49.7)	8646 (50.0)	35 (19.9)		8369 (52.2)	312 (21.9)	
Vitamin D, mean (SD)	27.38 (6.58)	27.38 (6.58)	27.29 (6.61)	0.847	27.46 (6.54)	26.49 (6.95)	<0.001
Vitamin D level, n (%)				0.923			<0.001
Normal	10227 (58.6)	10124 (58.6)	103 (58.5)		9501 (59.3)	726 (50.9)	
Low	5396 (30.9)	5343 (30.9)	53 (30.1)		4891 (30.5)	505 (35.4)	
Deficit	1835 (10.5)	1815 (10.5)	20 (11.4)		1639 (10.2)	196 (13.7)	
Osteocalcin, median [IQR]	15.35 [11.45, 21.03]	16.30 [11.75, 23.41]	15.35 [11.44, 21.01]	0.176	16.33 [11.62, 23.16]	15.29 [11.43, 20.85]	<0.001

^*^ Student’s t-test or Wilcoxon rank-sum test were used for continuous variables, and Chi-squared test was used for categorical variables.

IQR: interquartile range.

### Vitamin D levels – outpatient vs. inpatient

Vitamin D insufficiency was common in both cohorts. Outpatients had an overall mean 25(OH)D level of 26.25 ± 7.14 ng/mL (**Table 1**), with only about 48.0% of outpatients falling into the “Normal” vitamin D category (sufficient levels). The remaining outpatients had either low or deficient vitamin D status (approximately 52% combined). Outpatients had an overall mean 25(OH)D concentration of 26.25 ± 7.14 ng/mL, whereas inpatients had a slightly higher mean concentration of 27.38 ± 6.58 ng/mL. Nevertheless, low or deficient vitamin D status remained common in both cohorts, affecting 52.1% of outpatients and 41.4% of inpatients. Vitamin D deficit was noted in 14.0% of all outpatients and 10.5% of all inpatients. These findings indicate that whether in the community or hospital setting, a large proportion of patients had suboptimal vitamin D, underscoring a widespread issue.

### Vitamin D and arthritis

Among outpatients (**Table 1**), those with arthritis were significantly older than those without arthritis (mean age 49.20 vs. 41.15 years, *p* < 0.001) but had similar sex distribution (~81% female in both, *p* = 0.636). Mean serum 25(OH)D was slightly higher in the non-arthritis group (26.34 ± 7.10 ng/mL) compared to those with arthritis (25.60 ± 7.42 ng/mL), and this difference was statistically significant (*p* < 0.001). Notably, 16.2% of arthritis patients had a vitamin D deficit compared to 13.6% in non-arthritis patients. Log-binomial regression adjusting for age, sex, and osteocalcin showed that low vitamin D and vitamin D deficiency were associated with higher arthritis prevalence among outpatients compared with normal vitamin D (low: RR 1.24, 95% CI 1.14-1.34, p <0.001; deficit: RR 1.32, 95% CI 1.18-1.47, p <0.001, **Figure 1**).

**Figure 1:**
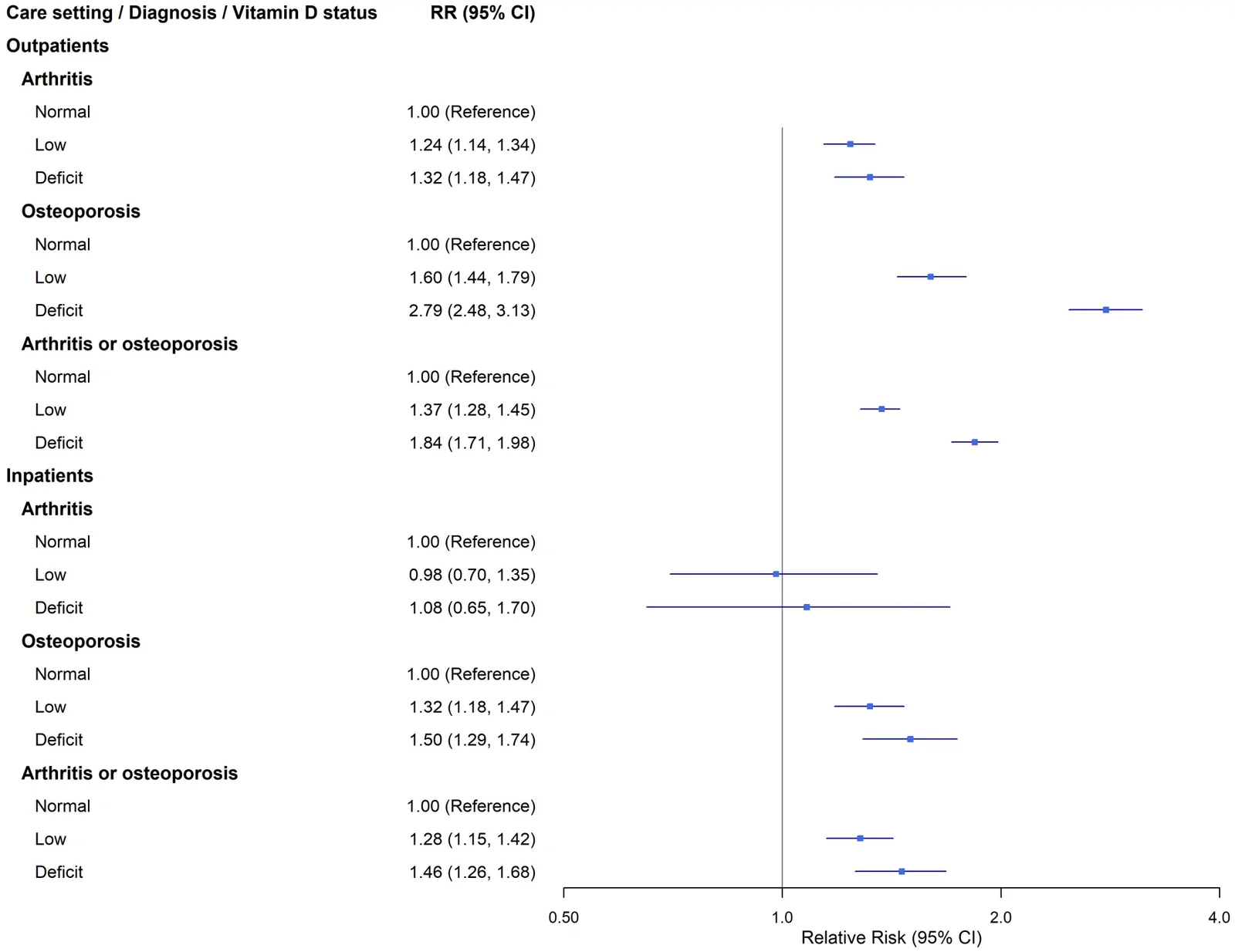
Adjusted relative risks (RRs) of arthritis, osteoporosis, and arthritis or osteoporosis by care setting and vitamin D status. Models were adjusted for age, sex, and osteocalcin; the alternate musculoskeletal diagnosis was additionally included in the separate inpatient models. Normal vitamin D was the reference category.

In the inpatient cohort (**Table 2**), the arthritis patients were notably older than other inpatients on average (65.32 vs. 57.70 years, *p* < 0.001) and much more often female (76.7% female vs. 42.8% in non-arthritis inpatients, *p* < 0.001), reflecting the female predominance of conditions like RA. Vitamin D levels did not differ significantly by arthritis status among inpatients. The mean 25(OH)D was 27.38 ± 6.58 ng/mL in non-arthritis patients versus 27.29 ± 6.61 ng/mL in those with arthritis (*p* = 0.847). The distribution of vitamin D categories was similar between the two inpatient groups: approximately 58% of both groups had normal vitamin D, around 30% had low levels, and 10–11% had a deficit (*p* = 0.923 for the overall distribution). However, only 176 inpatients had arthritis (1.0% of the inpatient cohort), and the adjusted estimates were imprecise (RR for vitamin D deficiency 1.08, 95% CI 0.65-1.70; RR for low vitamin D 0.98, 95% CI 0.70-1.35, **Figure 1**). Therefore, these inpatient data indicate no detectable association in this dataset, but they do not rule out a clinically meaningful association; the null finding should be interpreted in the context of limited power and wide confidence intervals.

### Vitamin D and osteoporosis

In the outpatient cohort (**Table 1**), as expected, individuals with osteoporosis were significantly older than non-osteoporotic outpatients (mean age 63.93 vs. 40.10 years, *p* < 0.001). Outpatient osteoporosis patients were predominantly female (84.4%), compared with 81.0% of those without osteoporosis (*p* < 0.001; a small but significant difference in sex distribution). The mean 25(OH)D level in non-osteoporotic outpatients was 26.50 ± 6.97 ng/mL, which was higher than the mean of 23.55 ± 8.34 ng/mL in those with osteoporosis (*p* < 0.001). Only 32.5% of outpatients with osteoporosis had normal vitamin D levels, in contrast to 49.4% of those without osteoporosis. Furthermore, 26.3% of osteoporotic patients had a vitamin D deficit, double the 12.8% observed in non-osteoporotic outpatients (Chi-square *p* < 0.001 for distribution). Consistently, adjusted risk analysis showed that vitamin D inadequacy in the outpatient setting was associated with a substantially higher risk of osteoporosis. Low vitamin D outpatients had 1.60 times the risk of osteoporosis (95% CI 1.44-1.79, p<0.001), and vitamin D deficit outpatients had 2.79 times the risk (95% CI 2.48-3.13, p<0.001), compared with those with normal vitamin D (**Figure 1**).

In the inpatient cohort (**Table 2**), patients with osteoporosis were largely elderly women: (87.7% female) with mean age 69.31 years. The mean 25(OH)D level of hospitalized non-osteoporosis patients was 27.46 ± 6.54 ng/mL, slightly but significantly higher than the 26.49 ± 6.95 ng/mL in osteoporotic inpatients (*p* < 0.001). Vitamin D sufficiency was less common in the osteoporotic inpatients: 50.9% had normal vitamin D, compared to 59.3% of those without osteoporosis, and more had a deficit (13.7% vs. 10.2%; *p* < 0.001 for the difference in distribution). Thus, even in the hospital setting, patients with osteoporosis tended to have worse vitamin D status than their counterparts, although the difference (an ~8–9 percentage-point gap in sufficiency rates) was not as dramatic as that seen in the outpatient setting. Adjusted analyses in inpatients confirmed a modest but significant elevation in risk: low vitamin D inpatients had a relative risk of 1.32 (95% CI 1.18–1.47, *p* < 0.001) for osteoporosis, and vitamin D deficit inpatients had a relative risk of 1.50 (95% CI 1.29–1.74, *p* < 0.001, **Figure 1**).

Osteocalcin concentrations were lower in the outpatient arthritis and osteoporosis groups and in inpatients with osteoporosis (**Tables 1**, **2**). The difference according to inpatient arthritis status was not statistically significant (**Table 2**).

The composite outcome of recorded arthritis or osteoporosis was present in 4,138 outpatients (20.3%) and 1,555 inpatients (8.9%). In the outpatient cohort, compared with normal vitamin D status, the adjusted RRs for the composite outcome (arthritis or osteoporosis) were 1.37 (95% CI 1.28–1.45) for low vitamin D and 1.84 (95% CI 1.71–1.98) for vitamin D deficiency. In the inpatient cohort, the corresponding adjusted RRs were 1.28 (95% CI 1.15–1.42) and 1.46 (95%CI 1.26–1.68), respectively (**Figure 1**).

In summary, our results demonstrate that vitamin D inadequacy was highly prevalent in this patient population, particularly among those with osteoporosis and, to a lesser extent, those with arthritis. Notably, patients with osteoporosis (in both outpatient and inpatient settings) had alarmingly high rates of vitamin D deficiency. Patients with arthritis also showed higher deficiency rates than those without in the outpatient setting; in inpatients, the arthritis subgroup was small and the estimates were imprecise, so the inpatient arthritis analysis should be viewed as inconclusive rather than as evidence of equivalence to patients without arthritis.

## Discussion

In this study, we investigated vitamin D status in large cohorts of outpatients and inpatients, comparing those with arthritis and osteoporosis to those without these conditions. The findings reveal a high burden of suboptimal vitamin D levels across the board, with particularly stark deficits in patients with osteoporosis. Vitamin D deficiency remained common among patients receiving clinical care. These results carry important public health implications, as vitamin D plays a vital role in maintaining bone health and possibly modulating chronic disease activity.

One of the key observations was that among outpatients, more than half of patients with arthritis or osteoporosis had low or deficient vitamin D levels. In the outpatient setting, 57.7% of arthritis patients and 67.5% of osteoporosis patients (**Table 1**) had less-than-normal vitamin D status (insufficient or deficient), despite being in ongoing care. This aligns with previous reports in the literature. For example, studies of RA patients have found vitamin D deficiency rates on the order of 43–84%, far higher than in the general population (35, 36). Low vitamin D in RA has been linked to greater disease severity and activity, as well as to poorer pain outcomes in some studies (37). A previous study in the U.S. reported that 57% of hospital inpatients were vitamin D deficient, although direct comparison is limited by differences in patient populations and deficiency thresholds (38). In such settings, having an arthritis diagnosis did not further stratify risk – because baseline risk was already high for all. In our study, the outpatient data clearly show that patients with chronic conditions like arthritis and osteoporosis are not achieving optimal vitamin D levels in the community. In contrast to the clear outpatient association, the inpatient arthritis analysis was statistically non-significant but underpowered because only 176 inpatients had arthritis. The wide confidence intervals mean that a clinically meaningful association cannot be excluded. Thus, the inpatient arthritis result is best interpreted as an absence of detectable evidence in this dataset, not as evidence that vitamin D status is unrelated to arthritis among hospitalized patients.

The clinical significance of these vitamin D disparities is considerable. In osteoporosis, vitamin D deficiency can directly worsen outcomes by impairing calcium absorption and accelerating bone loss (39). In our study, over one-quarter of osteoporotic outpatients had a vitamin D deficit. These patients are at heightened risk for osteomalacia and fractures if not addressed (40, 41). On the other hand, those osteoporosis patients who did have sufficient vitamin D likely represent the impact of osteoporosis clinics and public health messaging – a positive sign that intervention can work when implemented. Ensuring vitamin D sufficiency is known to improve response to osteoporosis treatments and may help reduce fracture rates (42). Similarly, for arthritis patients (especially RA), correcting vitamin D deficiency might have ancillary benefits on immune function and inflammation (43). Vitamin D has immunomodulatory effects, and deficiency in RA has been associated not only with higher disease activity but even with increased RA-related mortality in some cohorts (44). While our data do not directly address outcomes, they underscore that a large fraction of arthritis patients in routine practice have suboptimal vitamin D – a modifiable risk factor that clinicians could better manage.

Another noteworthy finding was the difference between outpatient and inpatient patterns. The vitamin D status of outpatients with chronic conditions was markedly worse relative to their counterparts without those conditions (45) (our study indicates that 26.3% vs 12.8% deficit in osteoporotic vs non-osteoporotic outpatients), whereas among inpatients the gaps were smaller or non-existent. This could suggest that once patients are hospitalized (for any reason), vitamin D deficiency is so prevalent that having a specific diagnosis like osteoporosis adds little additional risk; however, for inpatient arthritis, the more cautious interpretation is limited statistical power rather than a true absence of effect. It is also possible that inpatient care may include vitamin D supplementation in some cases (for example, hospitals often prescribe multivitamins or calcium/vitamin D to known osteoporosis patients during admissions), which could slightly improve levels in those patients. Outpatients, conversely, may only be tested for vitamin D if there is clinical suspicion (a selection bias), which might be why the overall outpatient cohort had a somewhat lower “normal vitamin D” percentage than inpatients. These considerations highlight the complexity of comparing settings. Nonetheless, the consistent theme is that vitamin D insufficiency is extremely common in both ambulatory and hospital contexts, and patients with bone and joint diseases remain especially vulnerable to deficiency.

The exploratory composite analysis conducted in the current study aimed to address the potential clinical overlap between arthritis and osteoporosis and variation in diagnostic recording, rather than to imply that the two conditions share a single pathophysiological mechanism. Low and deficient vitamin D status were associated with a higher prevalence of the composite outcome in both care settings. However, this analysis cannot identify clinically present conditions that were not recorded in the available diagnosis fields and should therefore be interpreted as an analysis of recorded diagnoses.

From a public health and clinical management perspective, our study’s findings reinforce the need for proactive strategies to improve vitamin D status among at-risk groups. It is not enough to assume that patients with known osteoporosis or arthritis are receiving appropriate supplementation; clearly, many are not reaching adequate levels. Below we outline key implications and recommendations:

Routine Screening and Monitoring: Healthcare providers should regularly screen 25(OH)D levels in patients with osteoporosis and inflammatory arthritis (like RA), both in outpatient clinics and upon hospital admission. Early identification of deficiency allows for timely intervention. Our data suggest that relying on self-reported supplement use or assumptions may miss a substantial proportion of deficient patients.

Targeted Supplementation: When deficiency or insufficiency is identified, vitamin D supplementation should be initiated and appropriately dosed. International and national guidelines support supplementation in these groups. For example, the International Osteoporosis Foundation recommends that seniors (≥60 years) take 800–1000 IU of vitamin D daily (46), and combined calcium–vitamin D supplementation is advised for individuals with osteoporosis (47). In patients with very low levels, higher therapeutic doses (e.g. 50,000 IU weekly or similar regimens for several weeks) may be required under medical supervision to replete stores (48). Ensuring adequate vitamin D (for instance, achieving serum 25(OH)D >30 ng/mL) is likely to improve bone density outcomes and might also help control disease activity in RA (49).

Education and Lifestyle Measures: Public health initiatives should aim to educate patients – especially older adults, those with limited sun exposure, and those with chronic diseases – about the importance of vitamin D. Dietary counseling (encouraging vitamin D–rich foods or fortified foods) and safe sunlight exposure advice can be given alongside supplements. Because many factors (season, latitude, skin pigmentation, age) affect endogenous vitamin D synthesis (50, 51), individualized advice is valuable. Patient education programs in rheumatology and geriatric clinics could improve supplement adherence, as non-adherence is a known barrier to effective vitamin D repletion.

Integrated Care in Hospitals: Hospitals and nursing facilities should incorporate vitamin D assessment into routine admission labs for high-risk patients (e.g. elderly individuals with fracture, RA patients on immunosuppressants, etc.). If deficiency is present, initiation of supplementation during the hospital stay can both begin repletion and signal the importance of follow-up to outpatient providers. Given the high prevalence of deficiency in acutely ill patients (as reflected in our inpatient cohort and prior studies (52)), treating this “silent” comorbidity could potentially improve patients’ recovery (e.g. muscle strength and mobility) and long-term musculoskeletal health (53).

### Strengths and limitations

This study has several important strengths. It includes a large sample with more than 37000 patients across both outpatient and inpatient settings, which provides strong precision for the overall cohort estimates and for the osteoporosis analyses. The inclusion of two key musculoskeletal conditions, arthritis and osteoporosis, allowed for broad yet detailed comparisons, and subgroup analyses helped clarify differences in vitamin D status across diagnostic and clinical care contexts. The use of real-world hospital data ensures that the findings are directly relevant to routine clinical practice, enhancing their applicability for public health and clinical decision-making.

Several limitations should be acknowledged. First, the data were derived from a single tertiary care center and may not be fully generalizable to other geographic regions or healthcare systems, particularly given differences in sunlight exposure, dietary habits, and cultural practices that can influence vitamin D status. Second, the “arthritis” category was heterogeneous and likely included a mixture of rheumatoid arthritis (RA), osteoarthritis (OA), and other arthritis subtypes, which may have distinct relationships with vitamin D deficiency. Because these subtypes could not be analyzed separately, caution is warranted when interpreting the pooled arthritis results. Another important limitation concerns differences in diagnostic data structure between care settings. Only one diagnosis field was available for outpatients, so coexisting arthritis and osteoporosis could not be identified and either condition may have been under-recorded when it was not the principal reason for the visit. Inpatients had up to four recorded diagnoses, permitting identification of some overlap, but clinically present conditions could still have been omitted. The composite analysis partly addresses variation in coding but does not eliminate under-ascertainment or diagnostic misclassification, which needs to be addressed by future studies with more comprehensive clinical data.

Third, information on vitamin D supplementation, calcium intake, and dietary habits was not available, limiting our ability to evaluate their potential influence on vitamin D status. In addition, diabetes mellitus data were available for inpatients but not consistently recorded in outpatient records. To maintain comparability across settings, diabetes was not included in the regression models, which may have resulted in residual confounding, particularly in outpatient analyses. Additionally, osteocalcin was included as a covariate to account for bone turnover; however, vitamin D-stratified analyses were not performed. Future studies incorporating more detailed bone metabolism assessments are needed to further clarify the interaction between vitamin D status and bone turnover markers. Furthermore, because several tables and multiple pairwise comparisons were evaluated, unadjusted p-values should be interpreted as exploratory and descriptive rather than confirmatory.

Another limitation is that the inpatient arthritis subgroup was relatively small (n = 176), resulting in wide confidence intervals. Therefore, the null findings in this subgroup should not be interpreted as definitive evidence of no association. Finally, the cross-sectional design limits causal inference, as exposures and outcomes were assessed at a single time point, preventing evaluation of temporal relationships, longitudinal changes, or disease progression. Seasonal variation in serum 25(OH)D levels was also not accounted for and may have influenced the observed results.

### Further directions

Our analysis was cross-sectional; longitudinal studies are needed to determine if improving vitamin D levels in these populations leads to measurable benefits (such as reduced fractures, improved arthritis control, or shorter hospital stays). Meanwhile our study shows a large age difference between osteoporosis patients and controls (mean 63.9 vs. 40.1 years). Although models adjusted for age, residual confounding may remain, which could affect the observed associations. Future studies using age-matched controls or additional adjustment methods are warranted to further address this issue. There is also a need for research into optimal implementation strategies – for instance, comparing different regimens of supplementation (daily low-dose vs. intermittent high-dose) in deficient patients with comorbid conditions. Additionally, investigating reasons for persistent deficiency (e.g. malabsorption, medication interactions, or patient non-adherence) would help tailor interventions. At the same time, the association between vitamin D and arthritis in outpatient versus inpatient settings was assessed separately rather than with a formal interaction term, so differences should be interpreted cautiously. Future studies with combined modeling are needed to confirm potential setting-specific effects.

## Conclusions

In conclusion, our research highlights a significant public health concern: insufficient vitamin D status is exceedingly common among patients with arthritis and osteoporosis, even among those engaged in medical care. Outpatients with these conditions showed higher rates of vitamin D deficiency than their counterparts without these diagnoses, and many hospitalized patients were also deficient. For inpatients with arthritis, the study found no statistically detectable association, but the small number of arthritis cases and wide confidence intervals mean that this result should be considered inconclusive rather than evidence of no effect. These findings underscore the need for systematic approaches to vitamin D assessment and management in high-risk populations. By improving vitamin D status in patients with osteoporosis and arthritis, we may reduce fracture incidence, enhance the effectiveness of osteoporosis treatments, and possibly ameliorate some aspects of arthritis disease activity. From a public health standpoint, tackling vitamin D deficiency in these groups, through screening, education, and appropriate supplementation, is a feasible and impactful strategy to support healthier aging and reduce the burden of musculoskeletal disease. Health authorities and policymakers should recognize the persistently high prevalence of vitamin D insufficiency and consider preventive strategies as a priority.

## Data Availability

The raw data supporting the conclusions of this article will be made available by the authors, without undue reservation.
